# Progress in preclinical studies of macrophage autophagy in the regulation of ALI/ARDS

**DOI:** 10.3389/fimmu.2022.922702

**Published:** 2022-08-18

**Authors:** Chang Liu, Kun Xiao, Lixin Xie

**Affiliations:** ^1^ School of Medicine, Nankai University, Tianjin, China; ^2^ College of Pulmonary & Critical Care Medicine, 8th Medical Center, Chinese PLA General Hospital, Beijing, China; ^3^ Medical School of Chinese PLA, Beijing, China

**Keywords:** macrophage, autophagy, acute lung injury, acute respiratory distress syndrome, treatment

## Abstract

Acute lung injury (ALI)/acute respiratory distress syndrome (ARDS) is a critical clinical syndrome with high morbidity and mortality that poses a major challenge in critical care medicine. The development of ALI/ARDS involves excessive inflammatory response, and macrophage autophagy plays an important role in regulating the inflammatory response in ALI/ARDS. In this paper, we review the effects of autophagy in regulating macrophage function, discuss the roles of macrophage autophagy in ALI/ARDS, and highlight drugs and other interventions that can modulate macrophage autophagy in ALI/ARDS to improve the understanding of the mechanism of macrophage autophagy in ALI/ARDS and provide new ideas and further research directions for the treatment of ALI/ARDS.

## 1 Introduction

### 1.1 ALI/ARDS

Acute lung injury (ALI)/acute respiratory distress syndrome (ARDS) was first proposed by Ashbaugh and colleagues in 1967 (1). Until 1994, ALI/ARDS was clinically defined as follows based on the definition put forward at the American-European consensus conference (2): patients with acute severe hypoxemia (ALI is diagnosed when PaO2/FiO2 > 200 mmHg and < 300 mmHg; the criteria for ARDS are met when PaO2/FiO2 < 200mmHg); chest radiography shows bilateral diffuse pulmonary infiltration; absence of increased pulmonary artery wedge pressure; and no clinical manifestation of left atrial hypertension. Subsequently, after the Berlin definition was proposed in 2012, the diagnostic criteria of ARDS were updated as follows (3): acute onset; chest imaging suggests bilateral infiltration that cannot be fully explained by exudation; lung lobar/lung collapse or nodules; respiratory failure that cannot be explicated by left heart failure or fluid overload; and when the minimum positive end expiratory pressure is 5 cm H2O, PaO2/FiO2 < 300 mmHg.

**Figure 1:**
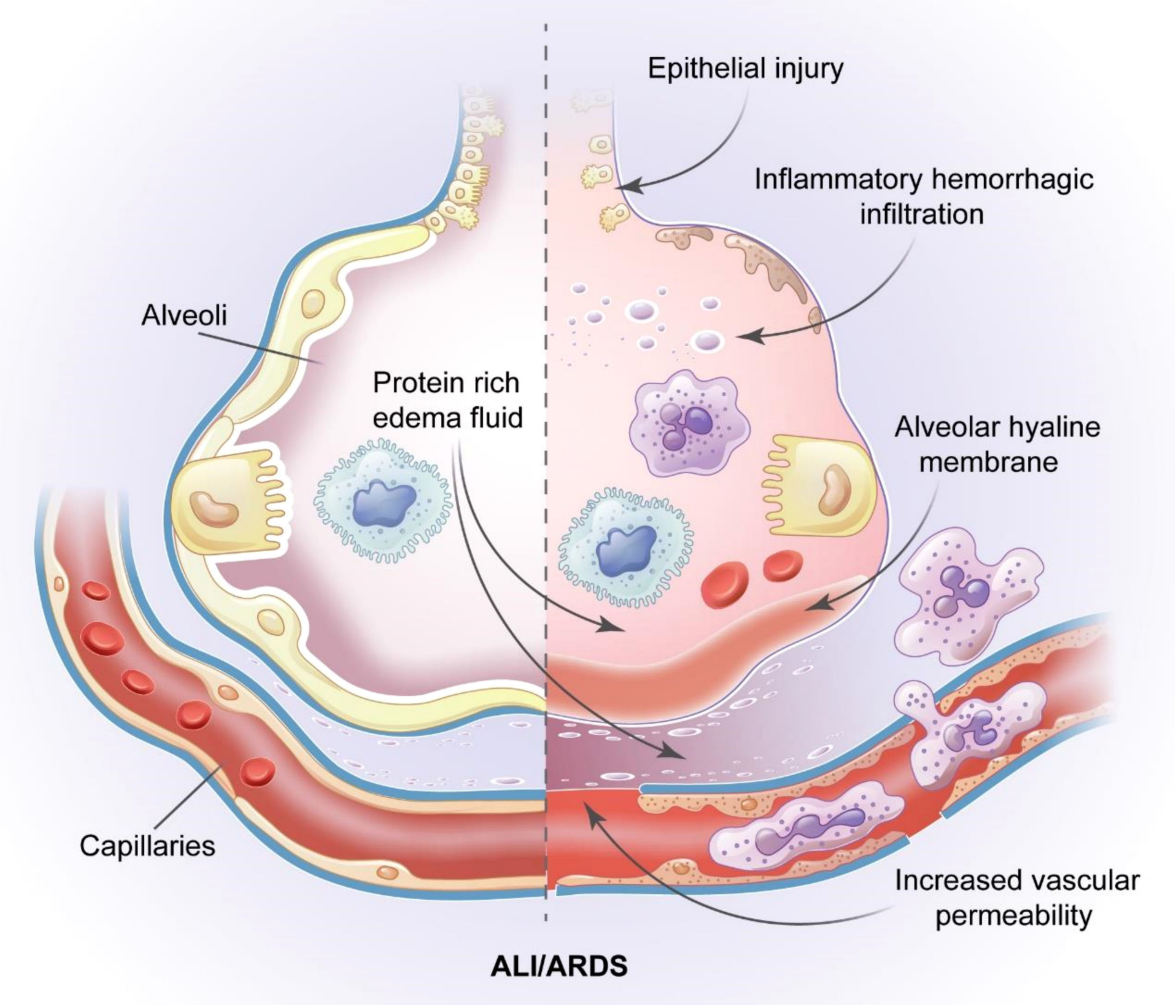
Pathological features of ALI/ARDS. The pathology of ALI/ARDS is characterized by diffuse alveolar capillary endothelial cells and epithelial cells necrosis, increased permeability of the pulmonary capillary endothelial cells and alveolar epithelial cell barriers, accumulation of protein rivh edema fluid, extensive pulmonary hemorrhagic changes and alveolar hyaline membrane and intracapillary thrombosis.

ALI/ARDS is a complex critical illness with the following main pathophysiological features: decreased pulmonary compliance; increased intrapulmonary shunt and physiological dead space; ventilation-perfusion imbalance; pulmonary edema caused by fluid exudation in the alveolar space; and increased alveolar capillary permeability. These features are caused by multiple non-cardiogenic intrapulmonary and extrapulmonary pathogenic factors (4). The main clinical manifestations of ALI/ARDS are the progressive exacerbation of intractable hypoxemia and hypoxic respiratory failure (5). The various causes of ALI/ARDS include aspiration, severe pneumonia, toxic inhalation, sepsis, trauma, fatty embolism, pancreatitis, and blood transfusion (6). The main feature of ALI/ARDS is diffuse alveolar damage, which is primarily caused by the necrosis and dysfunction of a large number of alveolar capillary endothelial cells and epithelial cells followed by the formation of a hyaline membrane and finally the establishment of intracapillary thrombosis (**Figure 1**) (7–9).

ALI/ARDS is an acute inflammatory reaction of the alveoli and pulmonary parenchyma accompanied by infiltration by inflammatory cells (e.g., neutrophils and macrophages) and alveolar hemorrhage. Neutrophils, which are the most abundant natural immune cells in human blood, play a key role in the pathogenesis of ALI/ARDS. After activation, neutrophils can release harmful mediators including cytokines, proteases, reactive oxygen species, and matrix metalloproteinases, leading to further damage (10–12). Some cytokines including IL-1, IL-6, IL-8, and TNF-α are pro-inflammatory factors that may aggravate lung injury.

In an international, multicenter, prospective cohort study on ARDS patients in intensive care units (ICUs) in 50 countries, Bellani et al. reported that the ICU incidence of ARDS was 10.4%, with 23.4% of ARDS patients requiring mechanical ventilation (13). In a prospective multicenter longitudinal study conducted in mainland China in 2020, Huang et al. reported that the prevalence of mild and severe ARDS patients was 9.7% and 47.4%, respectively (14). At present, there is no specific treatment for ALI/ARDS. The therapeutic approaches currently applied for ALI/ARDS include restrictive fluid management, mechanical ventilation, drugs including glucocorticoids and inhaled pulmonary vasodilators, and extracorporeal membrane oxygenation and other supportive treatments (4, 15, 16). Mechanical ventilation therapy for ALI/ARDS may involve pulmonary protective ventilation and prone position ventilation (17, 18). However, long-term mechanical ventilation treatment may lead to ventilator-associated events during prolonged hospital stays, increasing the risk of death (19). Although much progress has been made in the supportive treatment for ALI/ARDS, the mortality of ALI/ARDS patients remains high (35%–46%) (13). Even convalescent ARDS patients may have functional disabilities such as muscle weakness and fatigue after hospital discharge (20). Some patients may suffer from long-term neurocognitive impairment, psychological diseases such as depression or anxiety, and pulmonary insufficiency, leading to decreased quality of life (21, 22). Thus, ALI/ARDS is an urgent problem in the field of respiratory critical illness, and innovative mechanisms and therapies to alter the development and outcome of ALI/ARDS are urgently needed.

### 1.2 Autophagy

**Figure 2:**
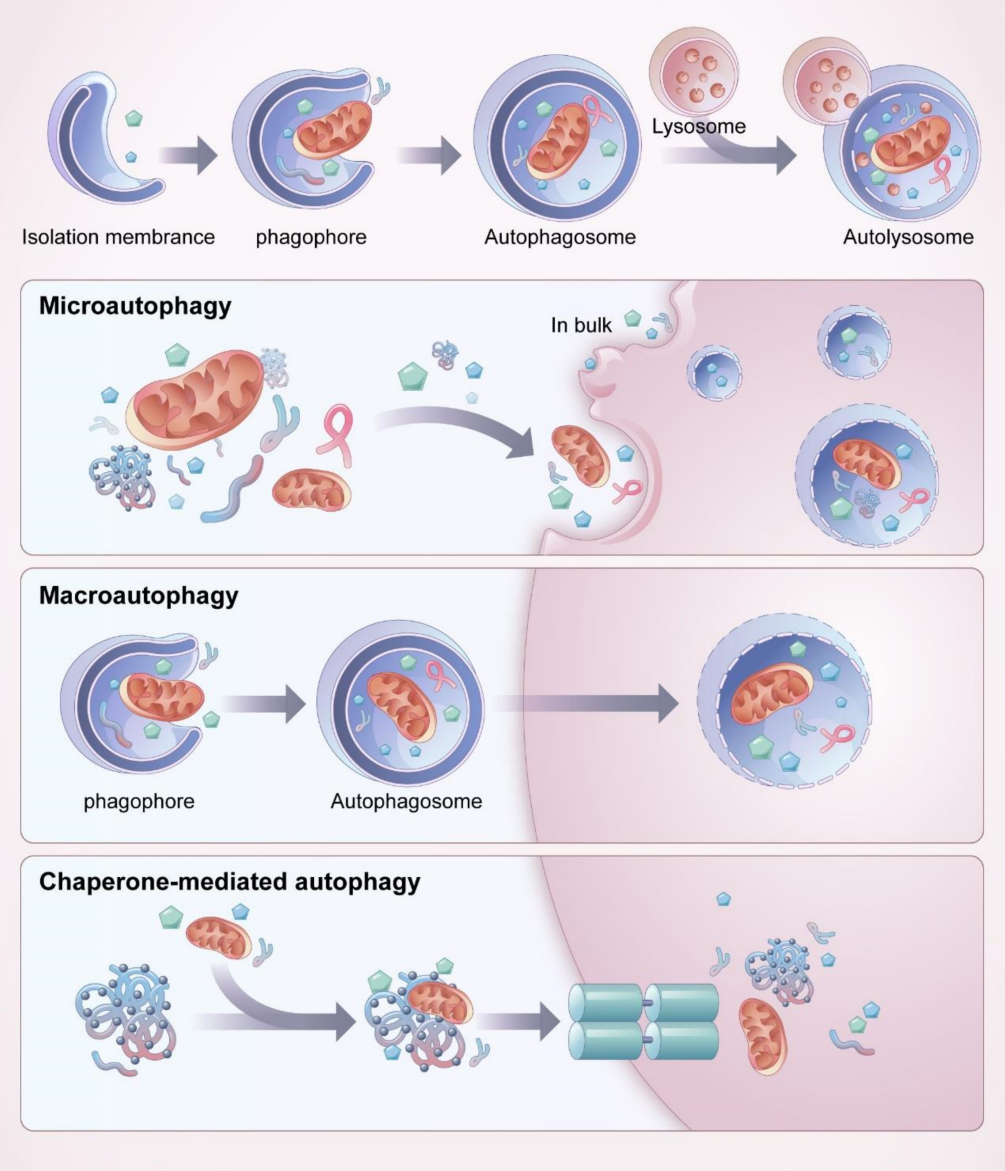
Process and classification of autophagy. Mitochondria and other organelles in the cytoplasm are firstly encapsulated by vesicles called “isolation membranes”, which gradually close to form a bilayer membrane structure, namely autophagosome. The outer membrane of autophagosome fuses with lysosome to form autolysosome, the contents and intima of autolysosome are degraded by enzymes in lysosomes. There are three main forms of autophagy, chaperone-mediated autophagy, microautophagy and macroautophagy, among which macroautophagy is the most common. Microautophagy directly wraps the substrate to be degraded into the lysosome for degradation through invagination or protrusion of the lysosomal membrane. In chaperone-mediated autophagy, a specific protein is involved, and the molecular chaperone recognizes the target protein through a specific structural domain and transports it into the lysosome for degradation. In macroautophagy, the autophaosome is required to wrap the substrate to be degraded.

Autophagy is a process of self-degradation that involves damage to organelles such as the mitochondria and endoplasmic reticulum, various pathogens, and abnormal proteins. Autophagy mainly occurs in eukaryotic cells and is generally considered to be the major adaptive response to maintain cell and tissue homeostasis under various stress states. Autophagy is closely associated with a variety of human diseases (23). Autophagy is an important mechanism by which cells adapt to changes in the external environment, maintain homeostasis in the internal environment, and resist invasion by foreign pathogens. There are three primary forms of autophagy (24), chaperone-mediated autophagy, microautophagy, and macroautophagy, with macroautophagy being the most common (**Figure 2**). In all three of these autophagy forms, damaged organelles or proteins are transported to the lysosomes for degradation and recycling. In microautophagy, the lysosomes directly encapsulate cytosolic components and transport them to the lumen of cytolytic organelles (25). In chaperone-mediated autophagy, the substrate reaches the lysosomal cavity directly through the protein translocation complex on the lysosomal membrane, independent of the capsule membrane or membrane invagination (26, 27). During macroautophagy, the cytoplasm and organelles are isolated in double-membrane vesicles, which transport the contents to lysosomes or vacuoles for degradation and recycling (28).

Autophagosome formation is a key step in autophagy and phagocytosis (**Figure 2**). During autophagy, isolation membranes are formed. The isolation membranes then nucleate, expand, and close to sequester cytoplasmic cargo. The isolation membranes with sequestered cargo then mature into autophagosomes, which are transported to the lysosomes, where they fuse with lysosomes or vacuoles. The contents of the autophagosomes are dissolved by various hydrolases contained in the lysosomes, and the degradation products are recycled for different cellular purposes (29, 30).

Autophagy involves proteins encoded by a series of related genes. Autophagosome formation is regulated by yeast Atg-related proteins (31, 32). The formation process of Atg12-Atg5-Atg16L promotes the formation of autophagosomes. First, ubiquitin-like protein Atg12 bonds with the lysine residue in Atg5 and then connects to Atg16L under catalysis by Atg10 to form the Atg12-Atg5-Atg16L complex, which is located on the outer surface of the isolation membrane to promote the extension and expansion of autophagy vesicles (33–35). LC3, a mammalian homologue of yeast Atg8p, is a crucial autophagy component and has been used as a specific marker of autophagy. After inducing autophagy, LC3I combines with phosphatidylethanolamine to form LC3II, which targets autophagic membranes. LC3II is stably retained on the autophagic membranes (36–39). Therefore, changes in LC3 localization have been used to measure autophagy.

### 1.3 Macrophages

Eile Metchnikoff first discovered macrophages in the late 19th century while observing the phagocytosis of pathogens during tissue inflammation (40). Macrophages are thought to be derived from bone marrow-derived monocytes, and these monocytes continue to be recruited into tissues and differentiate into tissue-resident macrophages (41, 42). However, numerous tissue-resident macrophages such as brain microglia, live Kupffer cells, cardic macrophages, large peritoneal macrophages and alveolar macrophages do not come from bone marrow-derived monocytes; instead, they are derived from embryonic yolk sac or fetal liver (43–48). Macrophages, which may have self-renewal potential similar to stem cells, remove pathogens and foreign bodies and proliferate in response to inflammation and other stimuli (49–51).

The innate immune system is the body ‘s first line of defense against external stimuli and includes macrophages, neutrophils, dendritic cells and natural killer cells. Autophagy has been proved to be closely related to the innate immune system and can alleviate excessive inflammatory responses (52, 53). Macrophages are important innate immune cells in the human body. The functions of macrophages include phagocytosis, antigen presentation, immune defense and immunomodulation, and the maintenance of tissue homeostasis (54–57). Macrophages are an important part of innate immunity, which is characterized by diversity and plasticity (58, 59). Macrophages can be divided into two polarization types: classically activated M1 macrophages and alternatively activated M2 macrophages (60). Macrophage polarization plays an important role in the development and regression of inflammation. M1 macrophages mainly secrete pro-inflammatory factors as part of the defensive immune response, while M2 macrophages principally secrete anti-inflammatory factors to promote tissue repair (60, 61).

Pulmonary macrophages exist for long time periods in the lungs, where they regulate the local pulmonary inflammatory microenvironment. Depending on anatomical location and function, the lung contains two different subpopulations of macrophages: alveolar macrophages (AMs) present in the alveolar lumen and interstitial macrophages (IMs) present in the interstitium (62, 63). AMs are the important innate immune cells and are located in the distal lung parenchyma of the alveolar cavity. These cells the first line of defense against foreign invasion, can initiate pulmonary immune response, and play a key role in maintaining the homeostasis of the pulmonary immune system (62, 63). AMs, including tissue-resident alveolar macrophages (TR-AMs) and monocyte-derived alveolar macrophages (Mo-AMs), have unique characteristics in both steady-state and diseases states (64, 65). TR-AMs play an important role in the removal of dead alveolar cells and excess alveolar surfactant (66, 67). In addition, TR-AMs are also sentineal cells that maintain immune homeostasis and play a crucial role in the regulation of pulmonary inflammation (68). TR-AMs can rapidly activate and release a variety of cytokines and chemokines after the onset of inflammation, because their surfaces contain various pathogen recognition receptors (68). Meanwhile, TR-AMs can also secrete a series of anti-inflammatory factors to promote inflammation regression and tissue repair (69). Moreover, TR-AMs also play a role in suppressing allergen-induced airway inflammation (70).

Mo-AMs are more susceptible to the lung microenvironment, and when injury occurs, monocytes reassemble in the alveolar lumen and differentiate into macrophages that cause tissue damage by releasing cytokines (71, 72). Thus Mo-AMs may be associated with the cytokine storm in severe infections. Lastly, Some researchers have identified two types of IMs, Lyve1loMHCIIhi IMs and Lyve1hiMHCIIloIMs. Lyve1loMHCIIhi IMs are mainly involved in inflammation and antigen presentation, while Lyve1hiMHCIIloIMs are mainly involved in wound healing and tissue repair (73).

These three types of macrophages interact with each other and together play a key role in immune surveillance and maintenance of immune homeostasis in the lung (74, 75).

## 2 Autophagy and macrophages

### 2.1 Autophagy regulates macrophage phagocytosis and antigen presentation

Macrophages, which are the key cells involved in immune response *in vivo*, are responsible for recognizing and clearing pathogens *via* phagocytosis. Macrophages and other immune cells use pattern recognition receptors to recognize invading pathogens by binding to pathogen-associated molecular patterns (76).

Autophagy affects phagocytosis by macrophages. Thus, a decline in autophagy function will affect macrophage phagocytosis. One study has Ganesan et al. reported that Salmonella typhimurium prevented autophagy by activating mammalian target of rapamycin (mTOR; the main autophagy inhibitor) in macrophages, which affected the phagocytosis ability of macrophages and led to a decline in S. typhimurium clearance (77). Mi et al. found infection with Listeria monocytogenes increased the mortality and level of serum pro-inflammatory factors in p38-regulated/activated protein kinase-deficient mice (78); the phagocytosis and bactericidal activity of macrophages in the mice were severely impaired, which may be associated with defects in autophagy induction. Moreover, Zhai et al. found that Mycobacterium tuberculosis could escape macrophage immune recognition and phagocytosis by inhibiting autophagy, resulting in latent infection (79).

Autophagy can also enhance the phagocytic function of macrophages. For instance, ABT-263, an inhibitor of the anti-apoptotic protein Bcl-2, enhances the bacterial phagocytosis of macrophages in aged mice by inducing beclin-1-dependent autophagy, which protects against sepsis (80). Xu et al. reported that autophagy promotes the phagocytosis of macrophages in Treponema pallidum *via* the nod-like receptor family domain containing 3 (NLRP3) inflammasome, the authors also found that macrophage phagocytosis was attenuated when transfecting siRNA targeting NLRP3 (81). LC3-associated phagocytosis is an unconventional autophagy-dependent process in macrophages. Inomata et al. demonstrated that macrophage LC3-associated phagocytosis is an immune defense mechanism against Streptococcus pneumoniae (82). While this defense could eliminate S. pneumoniae infection and regulate inflammation, it diminished with host age.

Macrophages can initiate and regulate the immune response. After engulfing pathogens and processing their antigens, macrophages migrate toward the T cells and stimulate them to resist microbial infection (83, 84). Activated macrophages express high levels of antigen-presenting molecules such as MHCI and MHCII on their surfaces (85). The MHC class II antigen presentation of macrophages is important for the recruitment of CD4+ helper T cells, which play an important role in the occurrence and development of cellular and humoral immune responses (86–89).

CCL-34, a TLR activator, can induce macrophage autophagy *via* the TLR4-NF-kB pathway, increase antigen processing, increase the antigen presentation of bone marrow-derived macrophages, induce the proliferation of antigen-specific CD4+T cells, and induce the production of activated T cell-related cytokines, IFN-γ and IL-2 (90). Zhang et al. reported that treatment with LPS increased the ratio of CD4+ to CD8+ T cells along with the expression levels of LCII and Beclin-1 in peritoneal macrophages (91). These findings indicate that macrophage autophagy plays a crucial role in regulating immune function in septic mice, and the mechanism may involve inflammation and macrophage antigen presentation.

Inducing macrophage autophagy can facilitate the clearance of pathogens *in vivo*. IFN-r can stimulate monocyte-derived macrophages in patients with cystic fibrosis, induce its autophagy, and increase antigen presentation, resulting in the enhanced clearance of Burkhloeria cenocepacia and a significantly reduction in bacterial load (92). Sengupta et al. demonstrated that rapamycin can promote macrophage autophagy to effectively eliminate Plasmodium during malaria infection (93). The enhanced autophagy improved the antigen presentation of spleen macrophages and strengthened T cell response. In contrast, the inhibition of autophagy may lead to bacterial immune escape. For example, the inhibition of autophagy by Mycobacterium tuberculosis PE_PGRS proteins decreased the presentation of MHCII-class restricted antigen, which provided immune escape for bacteria (94).

### 2.2 Autophagy regulates macrophage polarization

Macrophages, as heterogeneous cells, have a highly plastic response to various microenvironmental stimuli. Macrophages are polarized to the M1 phenotype in response to microbial stimuli such as lipopolysaccharide (LPS) Th1-related cytokines such as IFN-γ and TNF-α, activation of M2 macrophages is usually induced by IL-4, IL-13 and TGF-β (95–97). M1 macrophages release various pro-inflammatory factors and harmful mediators while clear pathogenic microorganisms, thereby aggravating tissue damage (98). M2 macrophages can release anti-inflammatory cytokines and inhibit the production of pro-inflammatory mediators, and remove apoptotic neutrophils from inflammatory sites to promote the repair of injury (98).

Autophagy, as a key component of cellular reprogramming, can facilitate the transition of macrophages from one phenotypic state to another (99). The degree of tissue inflammation caused by innate immune response depends largely on the balance between pro-inflammatory M1 and anti-inflammatory M2 macrophages. The immune homeostasis of the tissue microenvironment can be sustained by maintaining the balance of M1 and M2 macrophages in order to effectively avoid excessive inflammatory responses that cause tissue damage.

Autophagy regulates the inflammatory response by modulating M1/M2 macrophage phenotypic polarization. Autophagy can induce macrophages to polarize into the anti-inflammatory M2 phenotype. Docosahexanenoic acid is a kind of polyunsaturated fatty acid with anti-inflammatory effect on chronic inflammatory diseases and plays a key role in various inflammatory diseases including cardiovascular diseases and diabetes (100, 101). It can induce autophagy and enhance the expression of M2 macrophage markers (102). Laminaria japonica polysaccharide is one of the major natural active ingredients in Laminaria japonica, which can resist hyperglycemia, hypertension, hyperlipidemia and insulin resistance (103). Laminaria japonica polysaccharide decreased the expressions of M1 macrophage markers, increased the expressions of M2 macrophage markers, and reduced the degree of atherosclerosis damage in mice fed high-fat diets by enhancing the autophagic flux of macrophages, and this effect could be blocked by the autophagy inhibitor 3-methyladenine (3-MA) (104). In addition, TLR2-dependent autophagy can induce M2 macrophage polarization, regulate the NF-kB signaling pathway, and inhibit pro-inflammatory activity (105). High mobility group protein box 1 (HMGB1) is a member of the high mobility group protein family, and as the most characterized damage-associated molecular pattern (DAMP), it can trigger inflammation, innate and adaptive immune responses, and also tissue repair after injury (106, 107). In addition, hepatocellular carcinoma-derived high mobility group box 1 (HMGB1) can drive M2 macrophage polarization through TLR2-dependent autophagy (108). While blockade of HMGB1 reduced the accumulation of tumor-associated M2 macrophages and inhibited the growth of hepatocellular carcinoma in mice (108). Feeding with a high-fat diet can inhibit macrophage autophagy in mice. Macrophage autophagy deficiency may be the foundation of the inflammatory disease state, resulting in increased pro-inflammatory M1 macrophages and decreased anti-inflammatory M2 macrophages (109). Liu et al. demonstrated that autophagy deficiency enhanced the expressions of the pro-inflammatory mediators IL-1 β, IL-6, and TNF-α and promoted M1 macrophage polarization, which was manifested by increases in the expressions of surface markers iNOS and MCP1 and exacerbated acute liver injury (110).

However, autophagy has also been found to induce macrophage polarization to the M1 phenotype and inhibit M2 polarization. Advanced glycation end-products (AGEs) are a group of modified molecular products formed by nonenzymatic glycation reactions between carbonyl group of reducing sugars and the free amino group of proteins, lipids or nucleic acids, the formation and aggregation of AGEs can accelerate the progression of diabetic macroangiopathy by increasing intracellular oxidative stress (111, 112). Macrophage autophagy induced by AGEs promoted M1 macrophage polarization and hindered the healing of skin wounds (113). Adipose stem cell-derived exosomes promoted M2 macrophage polarization by inhibiting autophagy and significantly decreased the cerebral injury area of infarction (114). Isoprenaline promoted M2 macrophage polarization by downregulating autophagy, while the autophagy inducer rapamycin inhibited M2 polarization (115).

Zhao et al. reported that suppressing the mTOR signal promoted macrophage autophagy and inhibited M2 polarization, and this effect was eliminated by an autophagy inhibitor (116). In contrast, Zhang et al. found that inhibiting the mTOR signal activated autophagy cascades, increased the expressions of the macrophage surface markers Arg-1 and CD206, promoted M2 macrophage polarization, and played a protective role in atherosclerosis (117). These studies indicate that autophagy can modulate the secretion of inflammatory mediators and participate in the regulation of inflammatory response by regulating the polarization of macrophages.

## 3 Macrophage autophagy and ALI/ARDS

Autophagy plays different roles in the regulation of ALI/ARDS. Macrophage autophagy mainly reduces pulmonary inflammation and lung injury (**Table 1**). However, the effect of autophagy is not always a positive one. For example, macrophage autophagy has been shown to aggravate lung injury in some animal models (**Table 2**).

**Table 1:** Macrophage autophagy can reduce lung injury in animal models.

Authors	Publication time	Animal type	Injury model	Lung injury score	Factors regulating autophagy	Regulation pathway	Results	Reference
Jia et al	2019 Feb	BALB/c mice	LPS-induced ALI	16 (Edema, alveolar and interstitial inflammation, alveolar and interstitial hemorrhage, atelectasis, necrosis, and hyaline membrane formation were each scored on a 0- to 4-point scale)	Autophagy inducer-rapamycin; Enhanced autophagy in AMs.	Inhibition of NLRP3 inflammasome expression	LDH activity, total number of leukocytes, as well as neutrophils, macrophages and lymphocytes and MPO activity was significantly decreased in the rapamycin treatment group than the LPS group. Lung injury score was also decreased after rapamycin treatment.	(118)
Peng et al	2021 Jan	Male C57BL/6 mice	MTDs-induced ALI	3.5 (Lung injury scores were estimated by Smith’s scoring method, with a higher score indicating more severe injury)	Autophagy inducer-rapamycin; Enhanced autophagy in AMs.	Inhibition of NLRP3 inflammasome activation.	Lung injury score, the proportion of lung wet weight and the pulmonary capillary permeability, and the expression levels of IL-1β, TNF-α, and IL-18 proteins in BALF were decreased in the rapamycin treatment group than in the MTD-induced murine group.	(119)
Li et al	2021 Nov	C57BL/6 mice	CLP-induced ALI	10 (The lung injury of mice was assessed by the scoring system, which included the five parameters as follows: exudate, neutrophil infiltration, alveolar congestion, proteinaceous debris, and alveolar septal thickening.)	Knockdown of the GGPPS1 gene; Enhanced autophagy in AMs. Autophagy Inhibitor- 3-MA.	Induction of NLRP3 inflammasome inactivation.	The lung injury score of mice, the lung wet/dry weight ratio, the PaO2/FiO2 ratio, total protein content, total cell and PMNs counts were prominently increased in the CLP group compared with the sham group. 3-MA treatment further aggravated the above indicators. Silencing of GGPPS1 enhanced macrophage autophagy and induced the inactivation of NLRP3 inflammasome to relieve sepsis-induced lung injury.	(120)
Fan et al	2016 Dec	SD male rats	Lung ischemia-reperfusion injury	Not mentioned.	Autophagy inducer-rapamycin; Enhanced autophagy in AMs.	Reduction of endoplasmic reticulum stress levels in AMs	The percentage of TUNEL (+) cells (apoptosis cells) and the MDA activity was decreased in the rapamycin group than the control group, while the SOD levels were increased in the rapamycin group compared with the control group.	(121)
Liu et al.	2018 Dec	Male SD rats	LPS-induced ALI	14 (An ALI score was generated based on five independent features observed from HE images: neutrophils in the alveolar space, neutrophils in the interstitial space, hyaline membranes, proteinaceous debris filling the airspaces, and alveolar septal thickening.)	Lipoxin A4 receptor agonist BML-111; Enhanced autophagy in AMs.	Suppression of MAPK1 and MAPK8 signaling	The score of acute lung injury and lung wet/dry weight ratio were significantly higher in ALI rats than in BML-111 + ALI rats, suggesting that the prophylactic administration of BML-111 robustly alleviated ALI-associated lung injury.	(122)
Li et al.	2022 Jan	C57bl/6J male mice	CLP-induced ALI	10 (The severity of lung damage was scored according to bleeding, alveolar hyperemia, neutrophil infiltration, and alveolar dilatation.)	A novel H2S donor-GYY4137; Enhanced autophagy in AMs	Inhibition of mTOR signal pathway.	The animals’ survival rate, the lung injury score, the wet-to-dry lung weight ratio and SOD/MDA levels were significantly improved in GYY treatment group compared with CLP group.	(123)
Qu et al.	2019 Apr	Male BALB/c mice	LPS-induced ALI	2.8 (According to the degree of lung injury, bleeding, edema, exudation, necrosis, congestion, neutrophil infiltration, and atelectasis, they were evaluated on a scale of 0-4 points.)	Glycyrrhizic acid; Enhanced autophagy in AMs	Regulation of PI3K/AKT/mTOR signaling pathway	Lung weight coefficient, lung injury score, the levels of TNF-α, IL-1β were significantly decreased in GA+LPS group than LPS group, and these phenomena were reversed with 3-MA treatment.	(124)
Zhu et al.	2020 Sep	C57BL/6J mice	*P. aeruginosa*-induced ALI	5 (The degree of cellular infiltration was scored using previously described methods. The index was calculated by multiplying severity extent in 10 random areas, with a maximum possible score of 9)	A novel cobalquinone B derivative-CoB1; Enhanced autophagy in AMs	Regulation of AKT-mTOR signaling pathway.	The inflammation index, survival rate, bacterial burdens and inflammatory cytokines (TNF-α, IL-6, and IL-1β) were significantly decreased in PAO1+CoB1 group than PAO1+PBS group.	(125)

AMs, Alveolar macrophages; BALF, Bronchoalveolar lavage fluid; MTDs, Mitochondrial damage-associated molecular patterns; 3-MA, 3-Methyladenine; PAO1, The P. aeruginosa wild type (WT) strain.

**Table 2:** Macrophage autophagy can aggravate lung injury in animal models.

Authors	Publication time	Animal type	Injury model	Lung injury score	Factors regulating autophagy	Regulation pathway	Results	Reference
Hu et al.	2014 Jul	C57BL/6 male mice	Intestinal ischemia/reperfusion (IR) -induced lung injury.	2.5 (Lung injury was analyzed by an experienced investigator blinded for absent, mild, moderate, or severe injury (score 0–3) based on the presence of exudates, hyperemia and congestion, neutrophilic infiltrates, intra-alveolar hemorrhage and debris, and cellular hyperplasia.)	C5a bound to C5aR to activate AMs autophagy to exacerbate lung injury. Inhibition of autophagy through the autophagy inhibitor 3-MA or knockdown of the autophagy protein ATG5.	/	The pathological score and inflammatory cell infiltration was decreased in IR+C5a anti-body group than IR+C5a group. The inflammatory cytokines (TNF-α, IL-6, and MCP-1) was decreased in mice with Atg5-deficiency in macrophages than control group.	(126)
Yang et al.	2018 Jul	SPF C57BL/6 male mice	CLP-induced ALI	9 (Sections were stained with hematoxylin and eosin stain. The total score was calculated by adding up the individual scores of each category.)	Resveratrol (RSV); Inhibited autophagy in AMs.	Regulating the VEGF-B signaling pathway to inhibit the expression of C5aR	Lung injury score, MPO activity, albumin levels in BALF and levels of TNF-α, IL-6, and IL-1β were significantly reduced in RSV group than CLP group.	(127)
Liu et al.	2017 Apr	Bama minipigs	Lung I/R injury	Not mentioned.	HMGB1 and HSP60 could induce autophagy in AMs to exacerbate lung injury. Inhibition of autophagy by the autophagy inhibitor 3-MA or knockdown the autophagy proteins ATG7 and BECN1	Inhibition of TRAF6 ubiquitination and inhibition of MAPK and NF-kB signaling pathways.	Inflammatory cytokines IL-1β, TNF and IL12 were decreased in ATG knockdown group than the control group.	(128)

AMs, Alveolar macrophages; HMGB1, High mobility group protein box 1; HSP60, Heat-shocked protein 60; TRAF6, TNF receptor-associated factor 6.

### 3.1 Macrophage autophagy attenuates ALI/ARDS

#### 3.1.1 Macrophage autophagy reduces lung injury by inhibiting the formation and activity of the nod-like receptor family domain containing 3 (NLRP3) inflammasome

**Figure 3:**
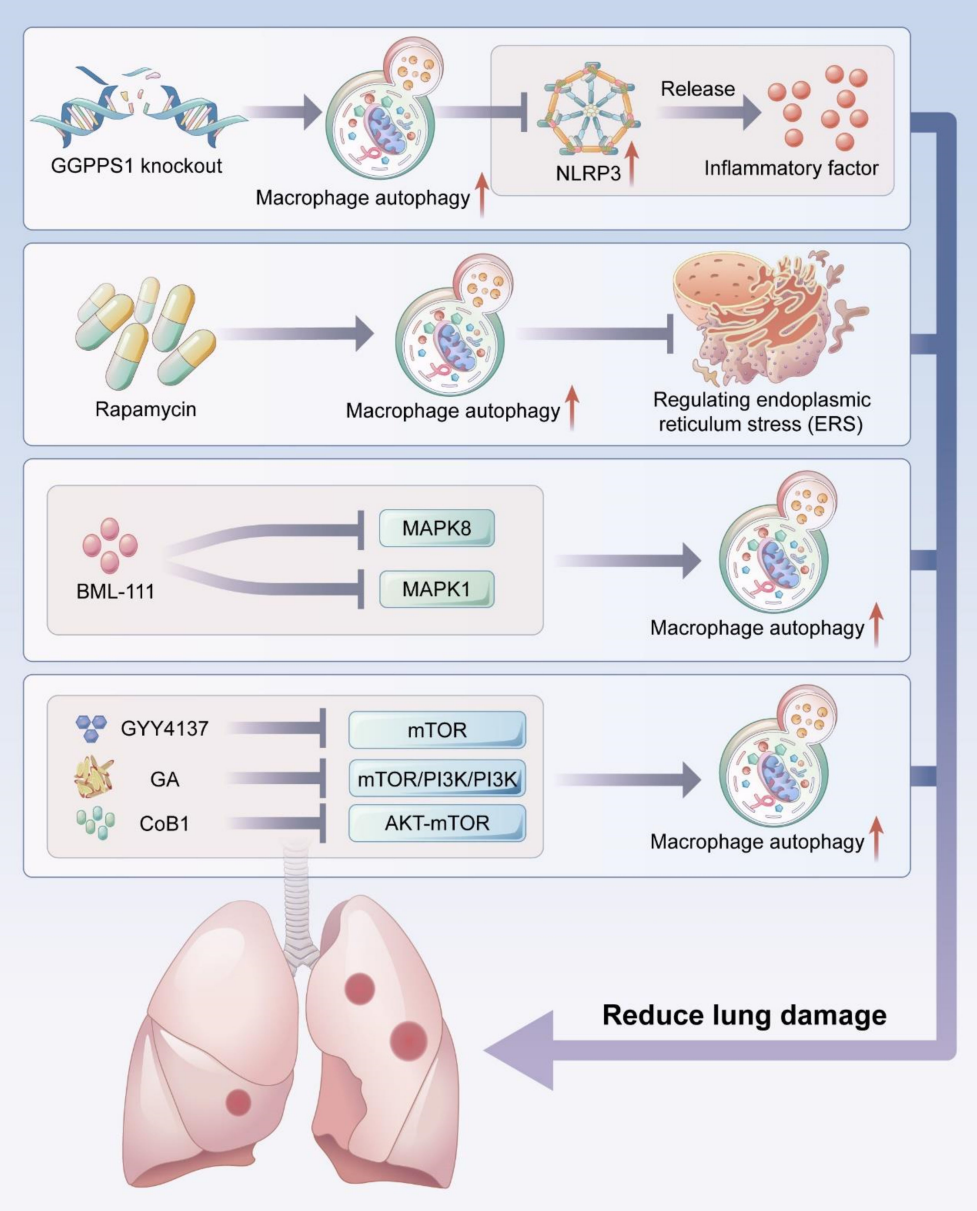
Macrophage autophagy reduces lung injury. Macrophage autophagy can reduce acute lung injury by inhibiting NLRP3 activity and endoplamic reticulum stress (ERS), and several studies have demonstrated that activating macrophage autophagy by targeting MAPK signaling pathway and mTOR signaling pathway can reduce lung injury.

The NLRP3 inflammasome is a multimeric cytosolic protein complex that serves as a key cytosolic innate immune signal receptor to sense pathogens. After its activation, NLRP3 inflammasome can mediate the secretion of a variety of pro-inflammatory cytokines to regulate inflammation-related disease pathogenesis (129, 130). Macrophage autophagy can attenuate pulmonary inflammatory response by inhibiting the formation and activity of NLRP3 inflammasome (**Figure 3**).

In the LPS-induced ALI model, the autophagy inducer rapamycin enhanced alveolar macrophage autophagy, inhibited the expression of NLRP3 inflammasome, reduced leukocyte infiltration, and decreased the secretion of inflammatory factors IL-1β and IL-18 in both pulmonary tissue and bronchoalveolar lavage fluid, which ameliorated the degree of lung injury (118).

Mitochondrial damage-associated molecular patterns (MTDs) are a type of damage-associated molecular patterns (DMAPs) that are released form mitochondrial rupture. MTDs can help trigger the inflammatory response and tissue injury by activating the pattern recognition receptors of immune cells (131). MTDs have been reported to induce NLRP3 inflammasome activation, resulting in severe inflammatory response in alveolar macrophages. Rapamycin, an autophagy inducer, can attenuate MTDs-induced inflammatory response by enhancing macrophage autophagy, reducing caspase-1 activation, inhibiting NLRP3 inflammasome activation, and suppressing the secretion of inflammatory cytokines such as IL-1β and IL-18 (119). However, the inhibition of autophagy by 3-MA exacerbated MTD-induced lung injury.

Geranylgeranyl diphosphate synthase 1 (GGPPS1) plays an important role in inflammation-related diseases (132). GGPPS1 expression was upregulated in the sepsis-induced mice lung injury model, and the activation of autophagy and NLRP3 inflammasome was found in the lung tissue of cecal ligation and puncture (CLP)-induced sepsis mice. The inhibition of autophagy aggravated hypoxemia, alveolar inflammatory response, and pulmonary edema; autophagy inhibition also significantly increased the lung injury scores, the expressions of inflammatory factors IL-1β and IL-18, and the expressions of NLRP3 and caspase-1 proteins in mice lung tissues. The knocking-down of GGPPS1 gene enhanced alveolar macrophage autophagy, as evidenced by an increase in the LCII/LCI ratio, decrease in p62 expression, and significant reduction of the expression of NLRP3 protein. These findings indicate that GGPPS1 knockdown can alleviate sepsis-induced lung injury by promoting autophagy to induce NLRP3 inflammasome inactivation (120).

#### 3.1.2 Macrophage autophagy reduces lung injury by regulating endoplasmic reticulum stress (ERS)

Intracellular homeostasis is disrupted when cells are stimulated by strong stimuli such as nutrient deficiency, calcium metabolic imbalance, and sustained oxidative stress. Such stimuli lead to the initiation of a series of cellular self-protective actions, including ERS. ERS refers to a perturbation of endoplasmic reticulum homeostasis that leads to the accumulation of unfolded or misfolded proteins in the endoplasmic reticulum lumen. ERS initiates the participation of unfolded proteins in the restoration of cellular proteostasis. When ERS is continuously activated, it can amplify the inflammatory response, induce cellular damage, lead to apoptosis, and accelerate the development of diseases (133–136). Fan et al. found that the autophagy promoter rapamycin significantly increased the protein levels of autophagy-related markers LC3, Beclin1, and HDAC6 and decreased the protein levels of apoptosis-related marker caspase-3 and ERS markers BIP, XBP-1, and CHOP in the early stage of lung ischemia-reperfusion injury (121). These findings suggested that autophagy could reduce the level of ERS in alveolar macrophages and decrease apoptosis, thereby maintaining local immune homeostasis in lung tissue to reduce lung injury (**Figure 3**).

In alveolar macrophages exposed to hypoxia-reoxygenation injury, the pre-treatment of alveolar macrophages with the proteasome inhibitor MG132 increased the expression of the autophagy marker LC3, suggesting that proteasome inhibitors an induce elevated levels of autophagy in alveolar macrophages (137). MG132 also led to the significant downregulation of ERS markers CHOP, BIP, and p-ERK and apoptosis-related protein caspase3/7 activity. These results suggested that MG132 could reduce lung injury by inducing autophagy and downregulating ERS in alveolar macrophages. Qian et al. demonstrated that enhancing autophagy in alveolar macrophages restored endoplasmic reticulum function in LPS-induced ALI, thereby hindering disease progression (138).

#### 3.1.3 Induction of macrophage autophagy by regulating mitogen-activated protein kinase (MAPK) pathway reduces lung injury

The MAPK signaling pathway regulates a variety of cellular processes and involves three main kinases: MAPK kinase kinase, MAPK kinase, and MAPK. The MAPK signaling pathway plays important roles in the regulation of cell growth, proliferation, differentiation, migration, apoptosis, and inflammation (139, 140). The MAPK signaling pathway is also involved in the progression of ALI/ARDS (141, 142). Treatment with the lipoxin A4 receptor agonist BML-111 increased the expressions of LC-II and Beclin1 and decreased the expressions of SQSTM1 and p62 in alveolar macrophages, suggesting that autophagy occurred in alveolar macrophages, and *in vivo* experiments confirmed autophagy reduced pulmonary histopathological damage and decreased lung wet/dry weight ratio. The levels of pro-inflammatory cytokines TNF-α and IL-6 in bronchoalveolar lavage fluid were also significantly decreased (122). The enhancement of autophagy was achieved *via* the suppression of the MAPK1 and MAPK8 signaling pathways, suggesting that BML-111 can induce autophagy in alveolar macrophages by targeting the MAPK pathway to reduce lung injury (**Figure 3**) (122).

#### 3.1.4 Induction of macrophage autophagy by regulating mammalian target of rapamycin (mTOR) signaling reduces lung injury

**Figure 4:**
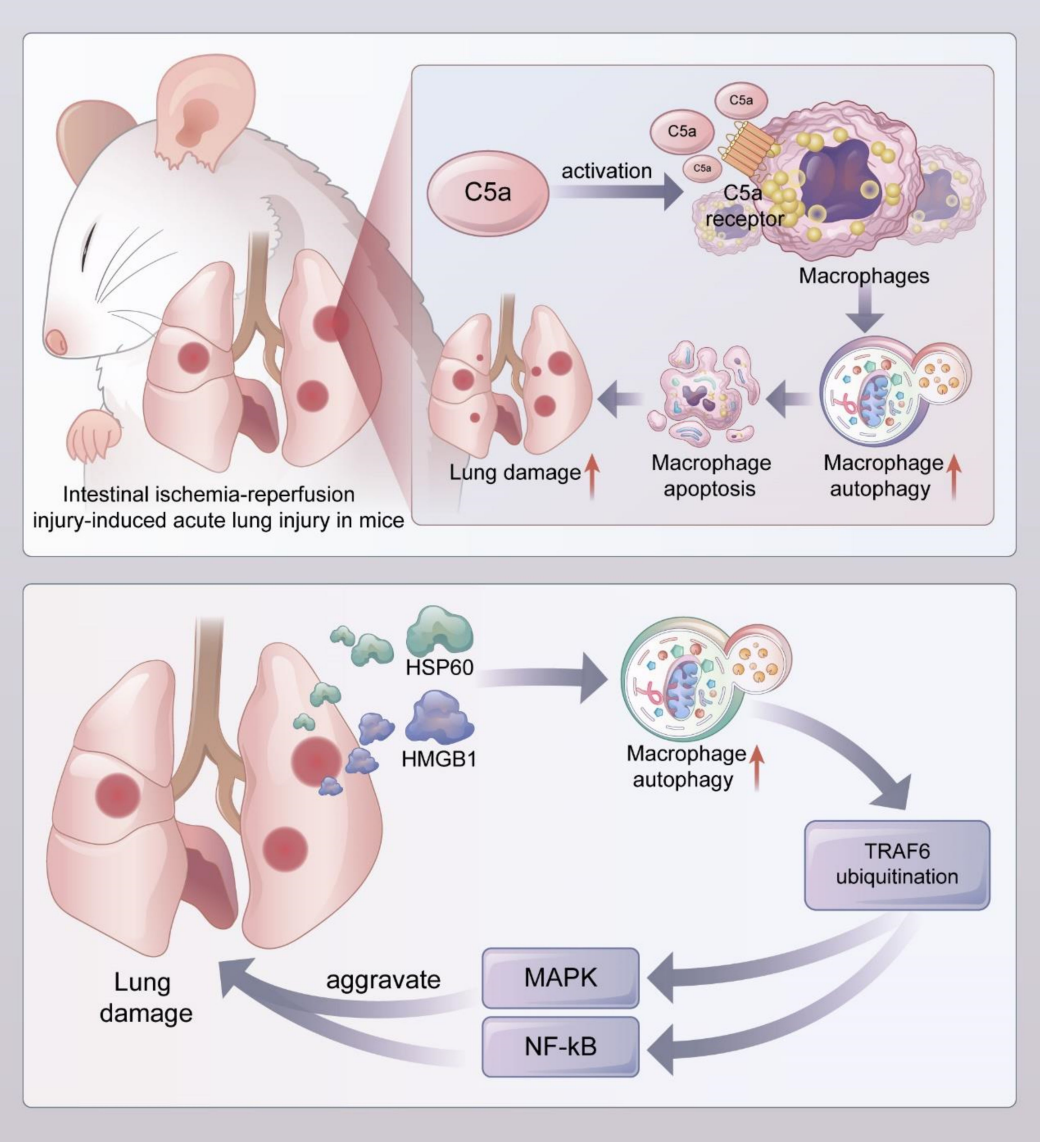
Macrophage autophagy aggravates lung injury **(** 1 **).** In the mouse model of acute lung injury induced by intestial ischemia-referfusion injury, complement C5a activated alveolar macrophages and bound to C5a receptors on their surface, leading to enhanced autophagy and induced apoptosis of alveolar macrophages, which exacerbated lung injury (2). DAMPs such as HMGB1 and HSP60 released during acute lung injury promoted uniquitination of TRAF6 by inducing autophagy of alveolar macrophages, and activated downstream MAPK and NF-kB signaling pathways to aggravate lung injury. DAMPs, Damaged-associated molecular patterns; HMGB1, High mobility group protein box 1; HSP60, heat-shock protein 60; TRAF6, TNF receptor associated factor 6.

The mTOR signaling pathway regulates the cell cycle, cell growth, and cell metabolism in physiological and pathological settings; it also plays a crucial role in lung injury (143–145). In an ALI model, LPS treatment significantly increased the expressions of p-mTOR, p62, and Beclin1, decreased LCII/LCI ratio expression, and enhanced the levels of inflammatory factors in macrophages (123). However, these effects were reversed after the administration of GYY4137, a novel H2S donor, suggesting that GYY4137 can improve autophagy and attenuate lung injury by blocking mTOR signaling (**Figure 4**).

mTOR is an important downstream target of the PI3K/AKT pathway with negative regulation of autophagy. Qu et al. demonstrated that treating macrophages with LPS increased the LCII/LCI ratio and Beclin-1 level and decreased p62 expression, indicating the activation of autophagy. Autophagy was further activated by treatment with glycyrrhizic acid (GA), and GA was one of the most important bioactive components of Glycyrrhiza uralensis with various effects such as immumodulatory activity and anti-inflammatory function (146). The protein levels of p-PI3K, p-AKT, and p-mTOR were inhibited by GA treatment. The ability of GA to induce autophagy in LPS-treated macrophages is at least partially attributed to the modulation of the PI3K/AKT/mTOR pathway (**Figure 4**). GA can reverse changes in pulmonary histopathological features *in vivo* by enhancing autophagy; for example, GA can reduce vascular congestion and bronchial wall thickening and suppressing the production of pro-inflammatory factors TNF-α and IL-1β, effects which can be reversed by 3-MA (124). Another study found that autophagy induced by the use of a novel cobalquinone B derivative (CoB1) in mice alveolar macrophages was associated with blocking the AKT-mTOR signaling pathway, thereby enhancing bacterial clearance and attenuating P. aeruginosa-induced lung injury (**Figure 3**) (125).

### 3.2 Macrophage autophagy aggravates ALI/ARDS

Autophagy in alveolar macrophages can exacerbate lung injury by inducing self-apoptosis. In animal models of lung injury, the complement activation product C5a has potent biological activity and can directly activate inflammatory cells such as neutrophils and macrophages to produce pro-inflammatory cytokines and chemokines that are involved in the progression of inflammatory diseases (147, 148). Sun et al. suggested that C5a produced during lung injury leads to apoptosis in alveolar macrophages by degrading bcl-2 after binding to C5a receptor (C5aR) (**Figure 4**) (149). In a mouse model of intestinal ischemia-reperfusion-induced ALI, complement C5a activated alveolar macrophages and bound to its surface C5a receptor, leading to the upregulation of LC-11 in alveolar macrophages, alveolar macrophage autophagy, and apoptosis, thus disrupting the lung dynamic equilibrium, and promoting the development of ALI/ARDS, while the injection of autophagy inhibitor 3-MA or knockdown of autophagy protein ATG5 in mice suppressed alveolar macrophage autophagy, inhibited macrophage apoptosis, and reduced the degree of lung injury (126). These phenomena suggest that the C5a-mediated autophagy of alveolar macrophages can induce macrophage apoptosis and thus promote the progression of lung injury (116). Yang et al. reported that macrophage autophagy levels were enhanced, and apoptosis was increased in an LPS-induced lung injury model compared to the control group (127). The treatment of alveolar macrophages with resveratrol, a potent SIRT-1 activator, decreased the level of autophagy, inhibited macrophage apoptosis, and significantly decreased LPS-induced C5aR gene expression; rapamycin reversed these effects (127). Qiu et al. reported that LPS-induced lung injury in rats was associated with self-apoptosis caused by the increased autophagy of alveolar macrophages; meanwhile, hydrogen-rich saline reduced apoptosis by inhibiting the autophagy of alveolar macrophages, thereby attenuating lung injury (150).

TRAF6 is a multifunctional adaptor protein that plays an important role in the induction of inflammatory response by activating the NF-kB and MAPK signaling pathways (151, 152). Alveolar macrophage autophagy can exacerbate lung injury by inducing TRAF6 ubiquitination. Liu et al. found that the release of damage-associated molecular patterns (DMAPs) such as HMGB1 and heat shock protein 60 (HSP60) from alveolar lavage fluid was significantly increased in lung ischemia-reperfusion injury, and the application of recombinant HMGB1 and HSP60 induced alveolar macrophage autophagy, as confirmed by the conversion of LCI to LCII, the upregulation of BECN1, and the degradation of SQSTM1, while the inhibition of autophagy by 3-MA or the knockdown of autophagy-associated proteins ATG7 and BECN1 in alveolar macrophages inhibited TRAF6 ubiquitination. Besides, the knockdown of ATG7 also decreased the phosphorylation levels of MAPK and NF-kB signaling activation markers in alveolar macrophages and significantly decreased the expressions of pro-inflammatory cytokines. Therefore, these results indicate that DAMPs released during lung injury can aggravate damage by inducing alveolar macrophage autophagy, promoting the ubiquitination of TRAF6, and activating the downstream MAPK and NF-kB signaling pathways (**Figure 4**) (128).

## 4 The modulation of macrophage autophagy is a promising strategy for reducing lung injury

Some drugs or interventions such as mesenchymal stem cells (MSCs) and MSCs-derived exosomes can reduce lung injury by regulating macrophage autophagy (153, 154). The G protein-coupled receptor cannabinoid receptor 2 agonist HU308 has been shown to enhance macrophage autophagy. In a mouse lung injury model, Liu et al. reported that HU308 reduced alveolar edema and inflammatory cell infiltration, alleviated hemorrhage and necrosis in lung tissue, and downregulated inflammatory factors, suggesting that HU308 can induce alveolar macrophage autophagy to reduce lung injury (155). The study also showed that inhibiting NLRP3 may be involved in the inhibitory effect of inflammatory responses by autophagy induction through cannabinoid receptor activation (155). Ying et al. reported that the inhibition of macrophage autophagy exacerbated inflammatory injury and inflammatory cytokine release, while Astragaloside IV induced macrophage autophagy by inhibiting the TLR4/NF-kB pathway, thereby reducing the inflammatory response and lung injury (156). The administration of NF-κB inhibitor further contributed to protein expression of autophagy, this suggested that the TLR4/NF-κB signaling pathway negatively regulates autophagy (156). Wang et al. found that the SIRT6 activator UBCS039 enhanced autophagy and M2 polarization in macrophages of a sepsis-induced ALI model, thereby reducing lung injury, while autophagy inhibitors eliminated this effect (157).The results found that SIRT6 overexpression restrained mTOR pathway activation, suggesting involvement of the mTOR pathway in SIRT6-regulated macrophage autophagy and M2 polarization (157). Yang et al. demonstrated that vitamin D induced macrophage autophagy and restored anti-inflammatory M2 macrophages in a silica-induced mice ALI model, thereby reducing inflammatory cell infiltration and mitigating lung injury (158). Vitamin D upregulated the Nrf2 signaling pathway, while depletion of autophagy related protein diminished the effect of vitamin D on regulating Nrf2 signaling pathway (158). Liu et al. reported that Buformin inhibited NLRP3-mediated pyroptosis in sepsis-induced lung injury by upregulating macrophage autophagy and Nrf2 protein through an AMPK-dependent pathway (159). The above results suggest that the modulation of macrophage autophagy by drugs may become an important target for mitigating lung injury.

MSCs and MSCs-derived exosomes also play key roles in regulating macrophage autophagy to attenuate lung injury. While moderate autophagy regulation is protective, over-active autophagy can lead to apoptosis or necrosis (160). Wang et al. found that oxygen glucose deprivation/restoration conditions resulted in a significant increase in the LC-II/LC-1 ratio and a decrease in p62 expression in alveolar macrophages accompanied by the upregulation of autophagy, while pretreatment with bone marrow mesenchymal stem cells (BMSCs) reduced macrophage autophagy and attenuated the ischemia/reperfusion-induced inflammatory response in ALI mice (153). BM-MSCs could modulate the autophagy of macrophage *via* thephosphoinositide 3-kinase/protein kinase B (PI3K/Akt) pathway and downstream signaling molecule heme oxygenase-1 (HO-1) (153). In addition, Liu et al. reported that the enrichment of miR-384-5p in BMSC-derived exosomes attenuated LPS-induced alveolar macrophage autophagy stress and alleviated LPS-induced alveolar macrophage apoptosis by targeting Beclin-1, reduced pulmonary vascular permeability and inflammatory response, and increased survival rate of ALI rats within seven days (154). MSCs-derived exosomes could alleviate LPS-induced ALI by reconstructing the miR-384-5p/Beclin-1 pathway (154). The molecular mechanisms of exosomal miRNA needs further investigation in the future.

However, the therapeutic intervention of targeted autophagy is still challenging to some extent. Although the mechanism of alveolar macrophage autophagy in ALI/ARDS have been increasingly investigated in recent studies, the specific mechanisms of macrophage autophagy remain unclear. At the same time, autophagy plays a double-edged sword role in lung injury. In addition to reducing lung injury, activation of autophagy can also exacerbate lung injury in some preclinical models, and therefore therapeutic measures targeting activation or inhibition of autophagy can have a positive effect in reducing ALI/ARDS caused by multiple etiologies. However, there are few successful examples of autophagy interventions successfully applied in clinical practice, and the application of therapies that promote insufficient autophagy or inhit excessive autophagy in clinical practice is still facing severe challenges, while how to clarify the changes of autophagy markers in ARDS patients is also a key point of carrying out clinical interventions targeting the modulation of alveolar macrophage autophagy.

At present, there are few clinical studies related to the treatment of ARDS patients by modulating autophagy, and more preclinical studies are still needed to confirm the potential beneficial role of regulating autophagy in ALI/ARDS. However, we found that some studies have confirmed the role of autophagy-related markers in the early identification and prognostic assessment of ARDS patients. For example, one study showed that the autophagy level of ARDS patients caused by sepsis was significantly inhibited. Moreover, the levels of autophagy-related proteins LC3II, Beclin-1, Rab7, Lysosomal Associated Membrane Protein 2 (LAMP2) and p62 had good value in the diagnosis and prognosis of ARDS patients due to sepsis (161). In addition, some investigators have found that increased levels of circulating mitochondrial DNA and activation of stimulator of interferon genes (STING) in patients with ALI/ARDS caused by sepsis (162). As an intracellular DNA sensing pattern recognition receptor that could cause damage to distant organs and lead to lysosomal acidification dysfunction, leading to autophagy dysfunction (163). Therefore, modulation of autophagy-related indicators may become a potential therapeutic approach for ARDS patients, and future clinical studies are still needed to demonstrate the beneficial effects of interventions to modulate autophagy in ARDS patients.

## 5 Conclusions

Autophagy is an important mechanism by which cells adapt to changes in the external environment and maintain the homeostasis of the internal environment. As a crucial component of innate immunity, macrophages play a key role in the regulation of inflammatory response and immune system homeostasis. Autophagy can regulate the functions of macrophages, including phagocytosis, antigen presentation, and polarization, and macrophage autophagy can have both positive and negative effects on the progression of ALI/ARDS. On one hand, autophagy can have a protective effect by removing harmful inflammatory factors from the body, thus reducing lung injury. On the other hand, autophagy can aggravate damage and lead to cell apoptosis, thus exacerbating lung injury. The regulation of macrophage autophagy is thus expected to be a promising approach for mitigating ALI/ARDS and lays the foundation for the discovery of novel drugs. In addition to regulatory measures targeting macrophage autophagy, modulation of other immune cells such as neutrophils and alveolar epithelial cell and endothelial cell autophagy within the lung tissue is also essential to reduce the symptoms of ALI/ARDS (164–167). Macrophage autophagy may be regulated by drugs actually used in clinical practice. For example, one study found that inhibition by corticosteroids and statins of macrophage autophagy enhanced IL-10 production, resulting in the control of asthmatic inflammation (168). In addition, another study found that simvastatin was shown to ameliorate asthmatic symptoms *via* autophagy augmentation (169). The autophagy of macrophages may be inhibited in COVID-19 patients. and the release of inflammatory cytokines caused by inhibition of macrophage autophagy may aggravate the cytokine storm (170, 171). Therefore, corticosteroids and statins may also play a role in COVID-19 by regulating macrophage autophagy. Vitamin D could also promote the production of antibacterial and antiviral proteins by macrophages, which inhibited the replication of viral particles and promoted the clearance of viruses from cells through autophagy (172, 173). Further studies on the action of these drugs in SARS-CoV-2 infection are necessary. However, most of the existing research findings come from animal and cellular experiments; the clinical application of therapeutic measures based on autophagy modulation remains a great challenge.

A variety of current drugs that activate or inhibit autophagy may lack specificity, such as low specificity for the target. For example, rapamycin selectively inhibits mTORC1, but long-term administration may promote the decomposition of mTORC2. It has revealed that alveolar macrophages are the dominant innate immune cells in the resolution of inflammation response and tissue repair through the influence on other immune cell populations in the lung. In addition, differences in the specificity of autophagy modulators may exist in that they non-selectively target a single cell type. Therefore, a full understanding of the regulation of autophagy acting on different cell types could help to develop modulators with better specificity. Finally, as there are various cell types that have distinctive functions in the lung, how to selectively target the autophagy process in a specific cell type without affecting the others remains to be an important issue.

## Author contributions

CL drafted the initial manuscript. KX and LX revised the manuscript. All authors contributed to the article and approved the submitted version.

## Funding

This work was supported by China PLA Scientific Key Grant (20-163-12-ZT-005-003-01), China Key Scientific Grant Program (No. 2021YFC0122500), National Key Research Program of China (2019YFC0121703), Key research project of basic plan (2019-JCJQ-ZD-117-01-3), National Science Foundation for Young Scientists of China (Grant No. 82100096) and National Science Foundation for Young Scientists of Beijing (Grant No. 7214254).

## Acknowledgments

The authors thank AiMi Academic Services (www.aimieditor.com) for the English language editing and review services.

## Conflict of interest

The authors declare that the research was conducted in the absence of any commercial or financial relationships that could be construed as a potential conflict of interest.

## Publisher’s note

All claims expressed in this article are solely those of the authors and do not necessarily represent those of their affiliated organizations, or those of the publisher, the editors and the reviewers. Any product that may be evaluated in this article, or claim that may be made by its manufacturer, is not guaranteed or endorsed by the publisher.
